# Social determinants, conflict, and displacement: reframing oral health in humanitarian action

**DOI:** 10.3389/fpubh.2025.1676346

**Published:** 2026-01-13

**Authors:** Elham Kateeb

**Affiliations:** 1 Oral Health Research and Promotion Unit, Al-Quds University, Jerusalem, Palestine; 2 World Dental Federation FDI Council, Geneva, Switzerland

**Keywords:** oral health, health policy, refugees, displaced persons, social determinants of health, humanitarian settings, health equity, universal health coverage (UHC)

## Abstract

Oral health is a fundamental component of overall health, yet it remains chronically excluded from humanitarian response frameworks. Displaced populations, including refugees and internally displaced persons (IDPs), experience a disproportionate burden of untreated oral disease, resulting in preventable pain, tooth loss, and systemic complications. Conflict and forced displacement intensify social determinants of health—transforming food insecurity, limited access to safe water, and the collapse of healthcare systems into acute risk factors for oral disease. Illustrative examples from diverse humanitarian contexts demonstrate how structural determinants such as malnutrition, attacks on health infrastructure, and psychosocial trauma converge to heighten oral health neglect. Despite well-established links between oral and systemic health, oral care remains absent from major humanitarian standards such as the Sphere guidelines and is underrepresented in UNHCR programming. Evidence also shows that low-cost, minimally invasive measures—such as daily oral hygiene support, fluoride use, and basic emergency dental interventions—are feasible even in fragile settings. This Perspective calls for urgent recognition of oral health as integral to humanitarian health policy and practice. Humanitarian actors, donors, and governments must ensure that oral health is systematically incorporated into emergency preparedness, response, and recovery. Integration with existing platforms—including maternal and child health, non-communicable disease services, and primary healthcare—offers practical and scalable entry points. Positioning oral health within humanitarian systems is both feasible and ethically imperative: a necessary step to relieve suffering, uphold dignity, and advance health equity for displaced populations worldwide.

## Introduction

Oral health, despite being a critical component of overall wellbeing, remains one of the most neglected areas in humanitarian response. This occurs even as oral diseases are among the most prevalent non-communicable conditions globally (1). In contexts of war, forced displacement, and natural disasters, oral healthcare infrastructure is often damaged or dismantled, leaving displaced populations without access to basic dental care.

This oversight is especially consequential given the well-established connections between oral health and systemic health outcomes, cardiovascular disease, diabetes, and adverse pregnancy outcomes (2). The continued exclusion of oral health from emergency response protocols and refugee health frameworks calls for urgent reconsideration.

Across conflict-affected regions, health inequities are amplified by structural determinants such as displacement, destruction of healthcare systems, resource scarcity, and loss of livelihoods. Conflict transforms conventional social determinants—like housing, education, food security, and sanitation—into acute health risks, particularly for oral health. Breaches of medical neutrality, social fragmentation, and psychosocial distress act as intermediaries linking conflict to worsening disease outcomes (3).

This Perspective draws on a narrative synthesis of current policy and public health literature regarding oral health in humanitarian settings. Targeted searches of PubMed and Google Scholar, complemented by WHO, UNHCR, UNRWA, and other authoritative sources were used to identify recent and policy-relevant evidence on oral health among conflict-affected and displaced populations. Examples are included from multiple global regions to illustrate how structural determinants translate into oral health outcomes and to highlight feasible models for integrating oral health into humanitarian systems. As a Perspective article, this work incorporates informed expert interpretation to identify policy gaps and future directions, rather than presenting findings from a systematic review.

## The oral health burden among displaced populations

The global displacement crisis has reached unprecedented levels. As of 2024, over 122 million individuals have been forcibly displaced, including 43.7 million refugees (4). Displaced populations face a disproportionate burden of oral disease due to precarious living conditions, nutritional deficiencies, and limited access to hygiene products and dental services. Evidence from both high- and low-income settings consistently shows that refugees experience worse oral health than host populations. For example, 85% of adult refugees in Canada had untreated dental decay compared to 20% in the general population (5). Similarly, 49% of refugee children in Massachusetts presented with untreated caries—more than twice the national average (6).

These vulnerabilities are further exacerbated in low-resource conflict affected settings. In Somalia, 60% of children suffer from dental caries and 70% from periodontal disease (9). In Gaza, prolonged malnutrition has led to vitamin C and calcium deficiencies, manifesting in gingivitis, bleeding gums, enamel erosion, and heightened susceptibility to infection (10). Disruptions in food and water supplies have intensified conditions like scurvy (10). In Ukraine, internally displaced persons had a mean DMFT index of 3.47, reflecting widespread untreated dental disease (11). Understanding these differentiated risks is essential for designing targeted, context-specific oral health interventions.

## Social determinants of oral health in conflict settings

Displaced populations face distinct yet equally severe barriers to oral healthcare access, shaped by structural determinants of power and marginalization (3). Internally displaced persons (IDPs), who remain within their country, often confront the complete collapse of infrastructure, restricted mobility, and legal invisibility (7). Refugees and asylum seekers may have access to healthcare services in host countries, but often experience limitations due to xenophobia, insufficient policy inclusion, and unfamiliarity with local systems (8).

In crisis settings such as the WHO Eastern Mediterranean Region—including the occupied Palestinian territory and Sudan, populations face what WHO terms a “double jeopardy”: chronic underdevelopment compounded by acute conflict. Essential determinants of oral health such as safe water, nutritious food, and sanitation are severely disrupted, while psychosocial distress exacerbates poor hygiene behaviors and neglect (3).

Conflict settings reshape the landscape of oral health by disrupting foundational determinants of wellbeing. The WHO highlights three unique social determinants in conflict zones—loss of human rights, breaches of medical neutrality, and the stress–disease progression pathway—which directly influence oral health through unmet basic needs, targeted damage to health systems, and stress-linked oral disease manifestations such as periodontitis, mucosal disorders, and temporomandibular dysfunction (3). These pathways underscore the need for oral health interventions that address both clinical needs and broader social disruption.

## Structural and operational deficiencies in humanitarian oral health response

Despite urgent need, oral health remains systematically excluded from major humanitarian strategies. A recent analysis of refugee health programs found virtually no integration of oral health into policy frameworks or service delivery mechanisms (12). This omission does not reflect infeasibility; rather, it arises from humanitarian prioritization models that continue to place oral health at the margins.

Oral health must be recognized and institutionalized as a vital component of primary healthcare within all humanitarian responses. Its integration is especially feasible and effective when linked with maternal and child health services or programs addressing noncommunicable diseases like diabetes. Such integration not only increases the accessibility and acceptability of oral health services but also facilitates smoother implementation across crisis settings. The global health community, including standard-setting bodies like SPHERE standards of humanitarian response (https://spherestandards.org/) and international agencies such as United Nations High Commissioner for Refugees UNHCR (https://www.unhcr.org/) must acknowledge this need explicitly in their policies and funding mechanisms (13).

In war-affected regions like Gaza, the collapse of health infrastructure has had devastating consequences. As of 2024, only 17% of primary healthcare facilities remained operational (14), and more than 435 attacks on health personnel were documented between October 2023 and April 2024, leading to a mass exodus and widespread psychological distress among medical professionals (15). Access to oral healthcare has been equally dire: by the summer of 2024, only 60 out of 1,500 licensed dentists were able to provide urgent care (10). Amid these extremely devastating conditions, some dental practitioners have been forced to deliver care from tents, using salvaged equipment and working with dwindling supplies (10). In such conditions, routine interventions such as restorative treatment, periodontal care, and preventive check-ups become unavailable. As a result, conditions that could normally be managed conservatively (e.g., with minimally invasive interventions) are left untreated until they progress, often forcing reliance on extractions or the prescription of antibiotics as the only viable options once pain becomes unbearable and infections advance.

Displacement further exacerbates the human resource crisis, which is compounded by systemic barriers such as economic hardship, language barriers, cultural stigma, and the psychological trauma experienced by refugees. Many displaced individuals are unfamiliar with, or deeply distrust, local healthcare systems due to previous encounters with institutional discrimination (25). In these circumstances, oral hygiene is often deprioritized in favor of immediate survival needs, perpetuating a cycle of neglect and preventable pain. These structural constraints accelerate the progression of preventable disease and deepen inequities in oral health outcomes.

## Pathways to integration: from emergency to sustainability

The integration of oral health into humanitarian response can be effectively achieved across three temporal phases. During the emergency phase, essential oral health services should be included within standard medical kits—such as silver diamine fluoride (SDF) for caries arrest, temporary restorations, infection-control supplies, and pain management. Equipping frontline healthcare workers with basic oral assessment skills can prevent complications and reduce downstream burdens on health systems (20).

In the post-emergency phase, collecting disaggregated data on oral health needs and strengthening community capacity through peer-led education can help reduce inequities. Teledentistry, already demonstrated as feasible in fragile contexts such as Sudan, can provide continuity of triage and consultation when physical infrastructure is damaged or inaccessible (21, 22).

Over the longer term, oral health should be fully embedded within universal health coverage (UHC) policies and national refugee health strategies. Integrating oral health into established platforms represents a scalable pathway to sustainable access and health equity. Examples from multiple regions illustrate that this integration is feasible even in resource-constrained settings. Hygiene kits in humanitarian distributions should include toothbrushes and fluoride toothpaste for daily prevention. Emergency dental kits for professionals—equipped with SDF, temporary restorative materials, extraction forceps, and anesthetic supplies—enable providers to deliver humane and effective care when comprehensive dental services are unavailable. Community-based models, including the United Nations Relief and Works Agency for Palestine Refugees in the Near East (UNRWA) school-based oral health programs (23, 24) and Chamas for Change in Kenya (19), show that prevention and culturally responsive delivery can achieve sustained improvements.

## Evidence-based interventions

Despite challenges, examples from different regions demonstrate that meaningful improvements in oral health are achievable even in fragile settings. In Brazil, Non-governmental Organizations NGO—health system collaborations in Manaus provided glass ionomer sealants and fluoride varnish for Venezuelan and Haitian refugees (16). In Bangladesh, culturally sensitive oral health education among Rohingya communities improved hygiene behaviors (26). In Ukraine and New Zealand, embedding oral health into emergency and settlement programs increased access to basic services and prevention (17, 18). Community-based initiatives such as the Chamas for Change program in Kenya have demonstrated sustainability and stakeholder ownership through culturally grounded prevention (19).

These examples collectively show that integration is both feasible and cost-effective when oral health is linked to trusted, existing systems of care.

This Perspective does not aim to catalog all humanitarian settings, but instead synthesizes illustrative examples to highlight shared challenges and policy opportunities. We acknowledge that environmental disasters and non-conflict crises warrant further examination. Ongoing initiatives, including an FDI World Dental Federation guide for oral healthcare delivery in emergencies, and a scoping review led by the authors on models of care in humanitarian settings—will provide more comprehensive evidence to support global policy action.

## Conclusion

Oral health in humanitarian settings is not a luxury—it is a fundamental component of health and human dignity. Yet its persistent exclusion from emergency response reflects not only gaps in service delivery but deeper structural inequities that marginalize displaced populations. Evidence from diverse humanitarian contexts demonstrates that even low-cost and minimally invasive interventions—such as daily toothbrushing support, fluoride toothpaste access, disease stabilization and emergency pain and infection control—are feasible and can prevent substantial suffering. Incorporating oral health into established humanitarian health platforms, including maternal and child health programs and primary care delivery, offers a pragmatic and scalable pathway forward.

Mainstreaming oral health within humanitarian policy and practice is therefore both a moral obligation and a practical imperative, fully aligned with global commitments to equity and universal health coverage. The opportunity to act is clear, the strategies are available, and improving oral health for displaced populations is an urgent step toward fairness, resilience, and comprehensive care.

## Data Availability

The original contributions presented in the study are included in the article/supplementary material, further inquiries can be directed to the corresponding author.
